# Involvement of *TRPC7-AS1* Expression in Hepatitis B Virus-Related Hepatocellular Carcinoma

**DOI:** 10.1155/2021/8114327

**Published:** 2021-08-31

**Authors:** Shaoliang Zhu, Hang Ye, Xiaojie Xu, Weiru Huang, Ziyu Peng, Yingyang Liao, Ningfu Peng

**Affiliations:** ^1^Department of Hepatobiliary Surgery, Guangxi Medical University Cancer Hospital, Nanning 530021, China; ^2^Department of Clinical Nutrition, Guangxi Medical University Cancer Hospital, Nanning 530021, China

## Abstract

**Objective:**

To investigate the expression of transient receptor potential (TRP) superfamily genes, especially *TRPC7-AS1* in hepatitis B virus- (HBV-) related hepatocellular carcinoma (HCC).

**Methods:**

Three cancer samples of HBV-related HCC at phase IV and matched paracancerous liver tissues were included in the study. Total RNA was extracted, and differential expression of RNA was screened by high-throughput transcriptome sequencing. The expression of *TRPC7-AS1* was detected by quantitative real-time PCR. The N6-adenosyl methylation RNA in MHCC97H, HepG2, and HL-7702 was enriched by coimmunoprecipitation with m6A antibody, and the relative level of N6-adenosyl methylation RNA in *TRPC7-AS1* was detected.

**Results:**

The expression of TRP family genes in cancer tissues was higher than that in paracancerous liver tissues, including *TRPC7-AS1*, *TRPC4AP*, *PKD1P6*, and *PKD1P1*. Moreover, the expression level of *TRPC7-AS1* in MHCC97H and HepG2 was also significantly higher than that in L02, a normal liver cell. The methylation level of N6-adenosine of *TRPC7-AS1* was lower in HepG2 cells than that in L02 cells.

**Conclusion:**

TRP superfamily genes, especially *TRPC7-AS1*, were highly expressed in HBV-related HCC. *TRPC7-AS1* could be a potential therapeutic target or diagnostic marker for HCC.

## 1. Introduction

Transient receptor potential (TRP) ion channel is a transmembrane protein, which plays key roles in mechanical injury, pain, temperature perception, and osmotic pressure perception by changing cell membrane potential or intracellular calcium concentration [1–4]. According to homology, the TRP ion channel family genes in mammals can be divided into six subgroups: TRP canonical (TRPC), TRP vanilloid (TRPV), TRP melastatin (TRPV), TRP ankyrin (TRPA), TRP mucolipin (TRPML), and TRP polycrystalline (TRPP). Among them, the first four subgroups belong to one class, and the latter two subgroups are classified as one group [5]. It has been reported that the dysfunction of the TRP ion channel (TRPV4, TRPV1, TRPM4, and TRPM) is considered to be related to obesity or diabetes, and these disorders are related to appetite, insulin secretion, and autoimmune response [6–10].

The downstream of the TRP ion channel family has a function in cell proliferation and is also considered to be related to cancer development (ref). TRPC6 was reported to be upregulated in glioblastoma, while TRPV2 was highly expressed in ovarian cancer [11, 12]. However, the involvement of TRP family genes in hepatocellular carcinoma (HCC) is still rarely reported.

In this study, the paracancerous tissues and the corresponding cancer tissues of three patients with stage IV hepatitis B virus- (HBV-) related HCC were taken as samples for high-throughput transcriptome sequencing. The expression levels of TRP family genes were analyzed, especially *TRPC7-AS1*. Additionally, the level of N6 adenosine methylation in HCC was also detected. This study would provide a potential therapeutic target or diagnostic marker for HBV-related HCC.

## 2. Materials and Methods

### 2.1. Patients

The paracancerous tissues and corresponding cancer tissues of three patients with phase IV HBV-related HCC were provided by Hepatobiliary Surgery, Cancer Hospital Affiliated to Guangxi Medical University. HepG2 cells (SCSP-510), HL-7702 cells (GNHu 6), and MHCC97H cells (SCSP-528) were provided by Cell Bank of Chinese Academy of Sciences (Shanghai, China). The study was approved by the Ethics Committee of Guangxi Medical University Cancer Hospital.

### 2.2. High-Throughput Sequencing

The paracancerous tissues and corresponding cancer tissues of three patients with stage IV HBV-related HCC were frozen, and the high-throughput sequencing was conducted by Shanghai Sangon Biotechnology Co., Ltd. (Shanghai, China) as previously described (ref).

### 2.3. Fluorescence Quantitative PCR

HepG2 cells, HL-7702 cells, and MHCC97H cells were cultured in DMEM supplied with 10% FBS (ThermoFisher, Massachusetts, USA). All cells were purchased from Wuhan Shangen Biotechnology Co., Ltd. (Wuhan, China). RNA was extracted from the cells in logarithmic growth using the ultrapure RNA extraction kit (CW0581M and CWBIO). After RNA was extracted, cDNA was synthesized according to the reverse transcription kit (CW2569M, CWBIO), and PCR reaction was carried out on the fluorescent quantitative PCR instrument with cDNA as template. The primers were synthesized by Shanghai Sangon Biotechnology Co., Ltd. (Shanghai, China). The primers included ENST00000514459F: 5′-GCCTCCTCCTTCCATAACG-3′, ENST00000514459R: 5′-CCCACAGCCTAGACCCATT-3'; GAPDH F: 5′-TGACTTCAACAGCGACACCCA-3′, and GAPDH R: 5′-TGACTTCAACAGCGACACCCA-3'.

### 2.4. RNA Coimmunoprecipitation (Co-IP)

With RNase inhibitors (cat.no), 5 × 10^6^ cells were lysed in 500 *μ*L lysate, and the lysate was used as the sample for Co-IP. 100 *μ*L of cell lysate was used as the input sample and another 100 *μ*L of cell lysate also used mouse lgG (provided by kit) for Co-IP experiment to get the IgG sample. Another 100 *μ*L cell lysate was incubated with m6A antibody (ab15123, Abcam, Cambridge, UK). The total RNA was extracted from the samples and reversely transcribed in the 50 *μ*L system to obtain cDNA. The cDNA was used as template, and *TRPC7-AS1* was used as the index for fluorescence quantitative PCR detection.

### 2.5. Statistical Analysis

All the data were presented as mean and standard deviation and analyzed by one-way ANOVA followed by the post hoc test by SPSS 19.0, with *P* < 0.05 as the significant difference.

## 3. Results

### 3.1. Expression Abundance of TRP Family Genes in HBV-Related HCC

**Figure 1:**
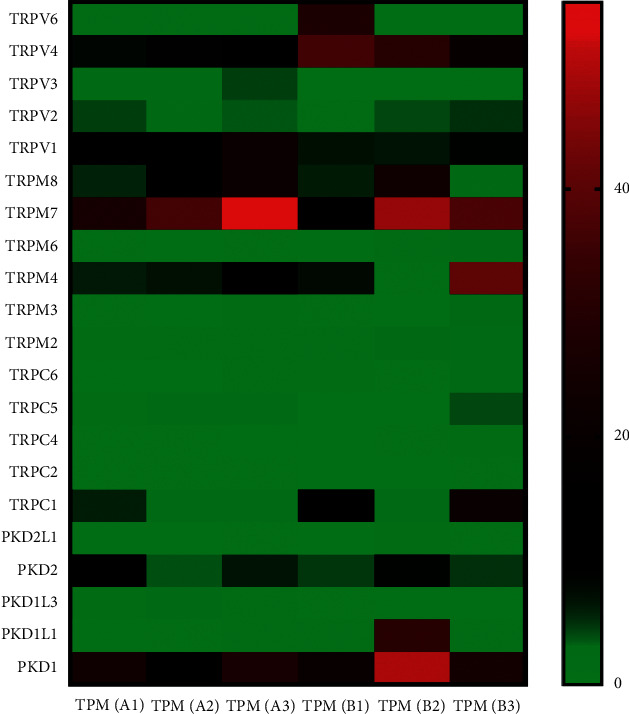
Expression abundance of TRP family genes in HBV-related HCC. Right side is the scale corresponding to the color and value. TPM, transcripts per million.

The paracancerous tissues (A1, A2, and A3) and the corresponding cancer tissues (B1, B2, and B3) of three patients with HBV-related HCC at stage IV were used as samples for high-throughput sequencing. The expression abundance of TRP family genes in the sample is shown in Figure 1. If TRP ion channel family genes were not detected in 4 or more samples, those genes are not shown in Figure 1, i.e., TRPC7 was not detected in 6 samples. The expression of TRP ion channel family genes in cancer tissue was higher than those in paracancerous tissue, and the expressions of TRPV6 (in B1 sample), TRPM4 (in B3 sample), TRPC1 (in B3 sample), and *PKD1P1* (in B2 sample) were significantly upregulated compared with those in paracancerous tissues.

The expression of TRP channel genes in cancer tissues was higher than those in paracancerous tissues.

The results of high-throughput sequencing showed that TRP ion channel-related genes, such as *TRPC7-AS1*, *TRPC4AP*, *PKD1P6*, and *PKD1P1*, were highly expressed in cancer tissues than those in paracancerous tissues (Table 1).

### 3.2. Expression Level of *TRPC7-AS1* in MHCC97H, HepG2, and L02 Cell Lines

**Figure 2:**
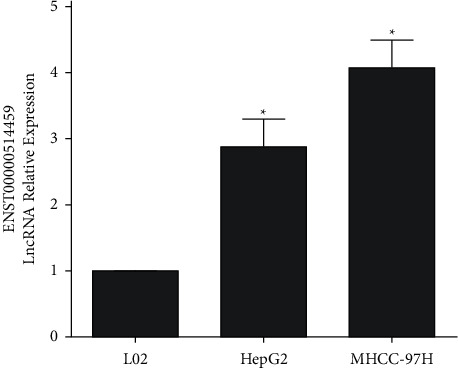
PCR results of *TRPC7-AS1* expression in L02, HepG2, and MHCC97H cells. Data are presented as mean and standard deviation. ^*∗*^*P* < 0.05 vs. L02.

The PCR results of *TRPC7-AS1* (transcript ID: enst0000514459) in MHCC97H, HepG2, and L02 cells are shown in Figure 2. MHCC97H and HepG2 were hepatoma cell lines, while L02 was normal cell line. Compared with L02 cells, the expression level of *TRPC7-AS1* in MHCC97H and HepG2 was significantly upregulated.

### 3.3. The level of N6-Adenosyl Methylation (m6A) of *TRPC7-AS1* in MHCC97H, HepG2, and L02 Cells

**Figure 3:**
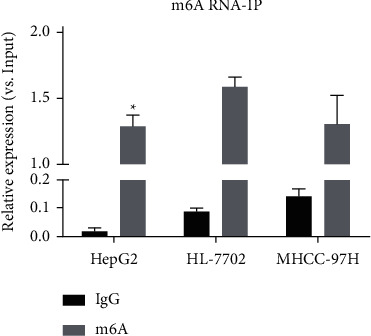
RNA Co-IP test results of the N6-adenosyl methylation (m6A) level of *TRPC7-AS1*. m6A: ^*∗*^*p* < 0.05 vs. L02 cells.

Through Co-IP with m6A antibody to enrich N6-adenosine methylation RNA in cells, the expression level of *TRPC7-AS1* was detected. Compared with the expression level of *TRPC7-AS1* in total RNA of the same amount of input samples, the relative level of N6 adenosine methylated in *TRPC7-AS1* was obtained (Figure 3, *P* < 0.05). The methylation level of N6 adenosine in *TRPC7-AS1* was lower in HepG2 cells than that in L02, but there was no significant difference between MHCC97H cells and L02 cells.

## 4. Discussion

In this study, we analyzed the expression abundance of TRP family genes in three cases of HBV-related HCC at stage IV. We found that the expression of TRP family genes in cancer tissues was higher than those in paracancerous tissues. In addition, we also found that TRP ion channel family genes (TRPV4, TRPV1, TRPM4, and TRPM5) related to obesity or diabetes were highly expressed in HBV-related HCC.

Compared with the paracancerous tissues, the high expression of TRPV4, low expression of TRPV1, and high expression of TRPM4 were found in HCC tissues, but TRPM 5 was not detected in 5 samples. The expression trends of TRPV1 and TRPM4 in the patients with diabetes are similar to the patients with obesity [6, 8]. TRPV4 has a regulatory effect on bodyweight and autoimmune inflammation, but whether it is positive or negative regulation is controversial [9, 10]. Diabetes is a risk factor of liver cancer, obesity, energy metabolism imbalance, and other states, which are indeed related to liver cancer [13]. Therefore, it can be inferred that the imbalance of TRP ion channel family gene expression is related to HCC.

We also found that the expression of TRP ion channel-related genes such as *TRPC7-AS1*, *TRPC4AP*, *PKD1P6*, and *PKD1P1* in cancer tissues was higher than those in adjacent tissues. Among them, the protein encoded by *TRPC4AP* gene is believed to be able to interact with TRPC ion channel and promote calcium release into cells, which is found to be related to Alzheimer's disease [14].

*TRPC7-AS1*, *PKD1P6*, and *PKD1P1* belong to lncRNA. More than 98% of the regions in human genome are noncoding regions. lncRNA is a kind of RNA which is widely transcribed but not translated to produce functional proteins. In recent years, lncRNA has been gradually found to play an important role in gene expression regulation [15]. *PKD1P6* and *PKD1P1* are pseudogenes of PKD1. Pseudogene is presumed to be a gene produced in the process of tandem doubling, gene mutation, or gene recombination of the parent gene without effective coding. In recent years, studies have found that pseudogene often has a regulatory effect on the parent gene, and the expression of pseudogene varies significantly in different cells, which may be supposed as the diagnostic and prognostic markers of cancer [16, 17]. The expression of *TRPC7-AS1* in the antisense chain of the intron region of TRPC7 gene was selected for further study. TRPC7 gene itself has not been detected in high-throughput sequencing of liver cancer samples, which may indicate that *TRPC7-AS1* cannot regulate the expression of TRPC7 gene in liver cancer. In this study, we found that the expression level of *TRPC7-AS1* in hepatoma cell lines was significantly higher than that in normal hepatoma cell lines, which was consistent with the high-throughput sequencing results of hepatitis B-related hepatoma samples. It was also found that the methylation level of N6-adenosine in *TRPC7-AS1* was higher in normal hepatocytes than that in hepatoma cells.

N6-adenosyl methylation (m6A) is the most common type of RNA modification, which is involved in the regulation of RNA cutting, transport, and degradation. For lncRNA, the increased m6A level often means poor stability of the structure [18, 19]. Moreover, the genes such as mettl3 and FTO which can regulate m6A have been also reported to be involved in the occurrence and development of cancer such as liver cancer [20, 21]. *TRPC7-AS1* has a low level of m6A in hepatoma cells, which is consistent with the high expression of *TRPC7-AS1* in hepatoma tissues and cells. It can be inferred that regulating the expression of m6A-related genes can play a role in regulating *TRPC7-AS1*.

In conclusion, the expression of TRP family genes in HCC and the correlation between *TRPC7-AS1* and HCC can be concluded by analyzing the experimental results of this study. In the future, we will select more target genes and carry out relevant functional experiments to explore the regulatory mechanism of *TRPC7-AS1* in the target genes.

## Figures and Tables

**Table 1:** The expression of TRP channel-related genes.

Gene name	Position	TPM (A1)	TPM (A2)	TPM (A3)	TPM (B1)	TPM (B2)	TPM (B3)	Gene description
*TRPC7-AS1*	5[+]136214048-136222159	0.00	0.00	0.00	24.65	1.98	6.89	TRPC7 antisense RNA 1

*TRPC4AP*	20[-]35002404-35092871	29.08	23.56	18.38	41.96	39.70	59.08	Transient receptor potential cation channel subfamily C member 4 associated protein

*PKD1P6*	16[-]15125242-15154564	14.68	12.48	15.99	17.64	43.34	22.32	Polycystin 1, transient receptor potential channel interacting pseudogene 6

*PKD1P1*	16[+]16310341-16334190	6.01	4.23	5.66	15.63	11.46	5.30	Polycystin 1, transient receptor potential channel interacting pseudogene 1 (source: HGNC Symbol; Acc: HGNC:30065)

## Data Availability

The data used to support the findings of this study are available from the corresponding author upon request.

## References

[1] Moran M. M. (2018). TRP channels as potential drug targets. *Annual Review of Pharmacology and Toxicology*.

[2] Nilius B., Appendino G. (2013). Spices: the savory and beneficial science of pungency. *Reviews of Physiology, Biochemistry & Pharmacology*.

[3] Patapoutian A., Tate S., Woolf C. J. (2009). Transient receptor potential channels: targeting pain at the source. *Nature Reviews Drug Discovery*.

[4] Guilak F., Leddy H. A., Liedtke W. (2010). Transient receptor potential vanilloid 4. *Annals of the new york Academy of Sciences*.

[5] Samanta A., Hughes T. E. T., Moiseenkova-Bell V. Y. (2018). Transient receptor potential (TRP) channels. *Subcellular Biochemistry*.

[6] Vennekens R., Nilius B., Colsoul B. (2013). Transient receptor potential (TRP) cation channels in diabetes. *Current Topics in Medicinal Chemistry*.

[7] Kyriazis G. A., Soundarapandian M. M., Tyrberg B. (2012). Sweet taste receptor signaling in beta cells mediates fructose-induced potentiation of glucose-stimulated insulin secretion. *Proceedings of the National Academy of Sciences of the united states of america*.

[8] Marshall N. J., Liang L., Bodkin J. (2013). A role for TRPV1 in influencing the onset of cardiovascular disease in obesity. *Hypertension*.

[9] O’Conor C. J., Griffin T. M., Liedtke W. (2012). Increased susceptibility of trpv4-deficient mice to obesity and obesity-induced osteoarthritis with very high-fat diet. *Annals of the Rheumatic Diseases*.

[10] Li Y., Sandra K., Jun W. (2012). TRPV4 is a regulator of adipose oxidative metabolism, inflammation, and energy homeostasis. *Cell*.

[11] Santoni G., Farfariello V. (2011). TRP channels and cancer: new targets for diagnosis and chemotherapy. *Endocrine, Metabolic & Immune Disorders - Drug Targets*.

[12] George S., Lehen’kyi V. ’y., Roman S. (2011). TRP channels in cell survival and cell death in normal and transformed cells. *Cell Calcium*.

[13] Wang P., Kang D., Cao W. (2012). Diabetes mellitus and risk of hepatocellular carcinoma: a systematic review and meta‐analysis. *Diabetes/metabolism Research & Reviews*.

[14] Poduslo S. E., Huang R., Huang J., Smith S. (2009). Genome screen of late-onset Alzheimer’s extended pedigrees identifies TRPC4AP by haplotype analysis. *American Journal of Medical Genetics Part B: Neuropsychiatric Genetics*.

[15] Run W., Yao Y. (2019). Cellular functions of long noncoding RNAs. *Nature Cell Biology*.

[16] Xiao-Jie L., Ai-Mei G., Li-Juan J., Jiang X. (2015). Pseudogene in cancer: real functions and promising signature. *Journal of Medical Genetics*.

[17] Laura P., Andrea M., Pier Paolo P. (2015). Pseudogenes in human cancer. *Frontiers of Medicine*.

[18] Wang X., Lu Z., Gomez A. (2014). N6-methyladenosine -dependent regulation of messenger RNA stability. *Nature*.

[19] Slobodin B., Han R., Calderone V. (2017). Transcription impacts the efficiency of mRNA translation via Co-transcriptional N6-adenosine methylation. *Cell*.

[20] Chen M., Wei L., Law C.-T. (2018). RNA N6-methyladenosine methyltransferase-like 3 promotes liver cancer progression through YTHDF2-dependent posttranscriptional silencing of SOCS2. *Hepatology*.

[21] Jia G., Fu Y., Zhao X. (2011). N6-Methyladenosine in nuclear RNA is a major substrate of the obesity-associated FTO. *Nature Chemical Biology*.

